# Unveiling Berberine’s Therapeutic Mechanisms Against Hepatocellular Carcinoma via Integrated Computational Biology and Machine Learning Approaches: AURKA and CDK1 as Principal Targets

**DOI:** 10.3390/ijms262110309

**Published:** 2025-10-23

**Authors:** Yuyang Wu, Yanmei Hu, Haicui Liu, Li Wan

**Affiliations:** School of Pharmacy, Chengdu University of Traditional Chinese Medicine, Chengdu 611137, China; yuyangwu@stu.cdutcm.edu.cn (Y.W.); huyanmei@stu.cdutcm.edu.cn (Y.H.); liuhaicui@stu.cdutcm.edu.cn (H.L.)

**Keywords:** berberine, hepatocellular carcinoma, AURKA, CDK1, machine learning, molecular docking, molecular dynamics simulation

## Abstract

Hepatocellular carcinoma continues to be a predominant contributor to oncological fatalities, characterized by restricted treatment alternatives. Although berberine exhibits anti-neoplastic capabilities, the underlying molecular pathways in hepatic malignancy require clarification. A comprehensive computational framework was established, incorporating transcriptomic data analysis, multiple machine learning methodologies, weighted gene co-expression network analysis (WGCNA), and molecular simulation techniques to elucidate berberine’s therapeutic pathways. Transcriptomic datasets from the Cancer Genome Atlas (TCGA) underwent examination to detect differentially expressed genes (DEGs). Ten machine learning methodologies screened critical targets, subsequently validated through molecular docking and 100 ns molecular dynamics simulations. Transcriptomic examination revealed 531 DEGs (341 exhibiting upregulation, 190 demonstrating downregulation) alongside 173 putative berberine interaction targets, yielding 17 intersecting candidates. Machine learning approaches consistently recognized AURKA and CDK1 as principal targets, subsequently confirmed by WGCNA as central genes. Elevated expression of both targets demonstrated correlation with unfavorable survival outcomes (*p* < 0.05). Computational docking analysis demonstrated robust binding interactions (AURKA: −8.2 kcal/mol; CDK1: −8.4 kcal/mol), with interaction stability validated through molecular dynamics simulations. Functional enrichment analysis unveiled targeting of cell cycle modulation, chromosome segregation, and p53 signaling networks. Berberine manifests anti-hepatocellular carcinoma activities primarily via coordinated targeting of AURKA and CDK1, essential cell cycle modulators. These discoveries provide molecular insights supporting berberine’s potential as adjunctive hepatic cancer therapy.

## 1. Introduction

Hepatocellular carcinoma represents one of the most challenging malignancies worldwide, ranking third in cancer-related deaths with a 5-year survival rate of only 18%. With over 800,000 new cases diagnosed annually worldwide [1], China accounts for approximately 400,000 diagnoses [2]. In China, primary liver malignancy constitutes the fifth most common cancer and second leading cause of tumor-related mortality. Although incidence rates are declining domestically, global forecasts predict a 55% increase in new liver cancer cases from 2020 to 2040, potentially resulting in 1.3 million deaths by 2040 [3]. While screening programs and vaccination initiatives show preventive promise, existing treatments like chemotherapy and radiotherapy face significant limitations including toxicity and resistance [4]. Therefore, developing more effective therapeutic agents with fewer side effects remains critical for improving patient outcomes.

**Figure 1:**
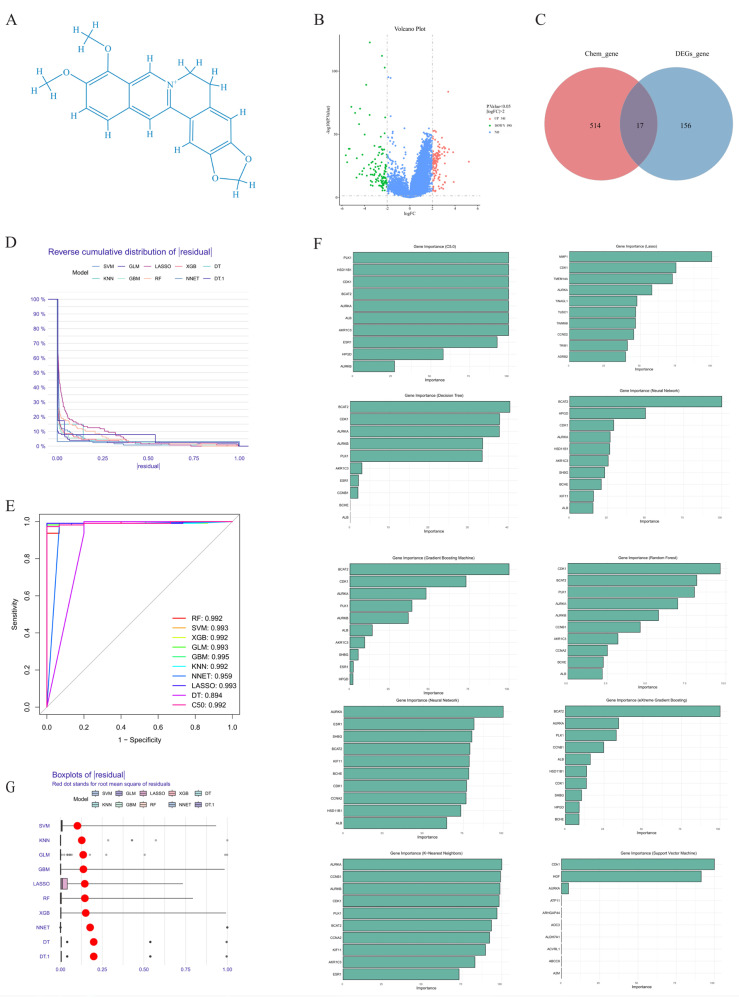
(**A**) Berberine structural formula. (**B**) Volcano plot displaying differentially expressed genes (DEGs) in hepatocellular carcinoma versus normal tissues. Red dots denote significantly upregulated genes, blue dots show downregulated genes, and black dots indicate non-significant changes. (**C**) Venn diagram showing intersecting target genes between berberine and hepatocel-lular carcinoma. (**D**) Inverse cumulative residual distribution across machine learning algorithms for PCB2 target gene prediction. (**E**) ROC curves evaluating predictive performance of various machine learning methods. (**F**) Gene feature importance rankings showing relative contributions to the predictive model. (**G**) Residual distribution box plots comparing algorithm performance.

Berberine (Figure 1A), an isoquinoline alkaloid derived from traditional Chinese medicinal plants such as Coptis chinensis and Phellodendron amurense, exhibits anti-cancer properties through multiple mechanisms [5]. These encompass cell cycle arrest, apoptotic induction [6], autophagy modulation [7], and radiosensitivity enhancement via Wnt/β-catenin pathway suppression [8], collectively suppressing hepatic cancer cell proliferation and inducing senescence. Combined therapies with 10-hydroxycamptothecin or regorafenib amplify treatment efficacy through topoisomerase inhibition [9], HIF-1α downregulation, or circular RNA modulation [10]. Despite extensive research spanning molecular to organismal levels, berberine’s precise anti-cancer mechanisms remain incompletely elucidated, particularly regarding cell cycle control and associated signaling networks. CDK1 (cyclin-dependent kinase 1) serves as a pivotal regulator in this context. Dysregulated CDK1 expression correlates strongly with hepatocarcinogenesis, disease staging, and prognosis. Research indicates CDK1 expression positively associates with PLK1, SGOL2, and ANLN genes [11]. CDK1 suppression disrupts cell cycle progression and diminishes PLK1, ANLN, and SGOL2 expression, highlighting the CDK1-PLK1/SGOL2/ANLN axis importance. Additionally, CDK1 coordinates mitochondrial metabolism through cyclin B1 interaction, synchronizing G2/M transition with cellular energetics [12]. AURKA (Aurora kinase A) is overexpressed in HCC, promoting tumor proliferation, metastasis, and invasion [13]. Through Myc [14], mTOR [15], and eIF4E pathway interactions [16], AURKA influences malignant characteristics, protein synthesis, and cell cycle control. Berberine potentially inhibits AURKA activity, consequently disrupting proliferative processes.

Contemporary computational biology integrates multi-omics data to illuminate cancer mechanisms [17], identifying differentially expressed genes and berberine interaction targets. Machine learning algorithms efficiently screen critical features from these datasets [18], advancing therapeutic understanding [19]. Weighted Gene Co-expression Network Analysis (WGCNA) reveals co-expressed gene modules and hub genes, unveiling regulatory networks, while Protein–Protein Interaction (PPI) analysis combined with machine learning identifies key therapeutic targets [20]. Computational chemistry advances have enhanced molecular docking and dynamics simulations in pharmaceutical research [21], predicting ligand-binding conformations and revealing interaction networks [22]. These methods validated berberine-target associations. This integrated approach combining computational biology, machine learning, WGCNA, and simulations elucidated berberine’s hepatic cancer mechanisms, providing drug development insights. Therefore, this study employed an integrated computational biology and machine learning approach to elucidate berberine’s mechanisms, identifying AURKA and CDK1 as its principal therapeutic targets against hepatocellular carcinoma.

## 2. Results

### 2.1. Prediction of Biological Targets of Berberine in Liver Cancer Treatment

Volcano plots revealed gene expression changes and statistical significance, characterizing differentially expressed gene (DEG) patterns. Analysis identified 531 DEGs: 341 upregulated and 190 downregulated (Figure 1B). SwissTargetPrediction and PharmMapper predicted 173 berberine target proteins. Intersection analysis with hepatic cancer DEGs yielded 17 overlapping proteins (Figure 1C). These overlapping targets represent potential therapeutic nodes linking hepatic cancer pathogenesis with berberine’s pharmacological effects, suggesting berberine’s therapeutic potential in hepatocellular carcinoma.

### 2.2. Core Target Proteins Screened by Machine Learning

All algorithms exhibited robust predictive performance for identifying AURKA and CDK1 as target genes. Cumulative distribution curves of residuals were smooth, with proportions decreasing gradually as residual values increased, indicating high prediction accuracy (Figure 1D). Receiver Operating Characteristic (ROC) curves (Figure 1E) further revealed that, except for the Decision Tree (DT) algorithm with an Area Under the Curve (AUC) of 0.894, the AUC values of the remaining nine algorithms exceeded 0.9. This demonstrates consistent predictive capabilities across different models despite minor performance variations. The AUC metric, by reflecting ranking accuracy across classification thresholds, further validates the reliability of machine learning-based target gene screening. During model training, we also calculated feature importance to evaluate the contribution of individual genes to model predictions. The results showed that AURKA and CDK1 ranked prominently in the feature importance list, further confirming the robustness of the screening results (Figure 1F). To visualize the distribution of prediction residuals more intuitively, we generated box plots. These plots revealed that the residual distributions across different algorithms were relatively compact, with medians close to zero, indicating significant stability and consistency in the prediction results (Figure 1G).

### 2.3. Weighted Gene Co-Expression Network Construction

**Figure 2:**
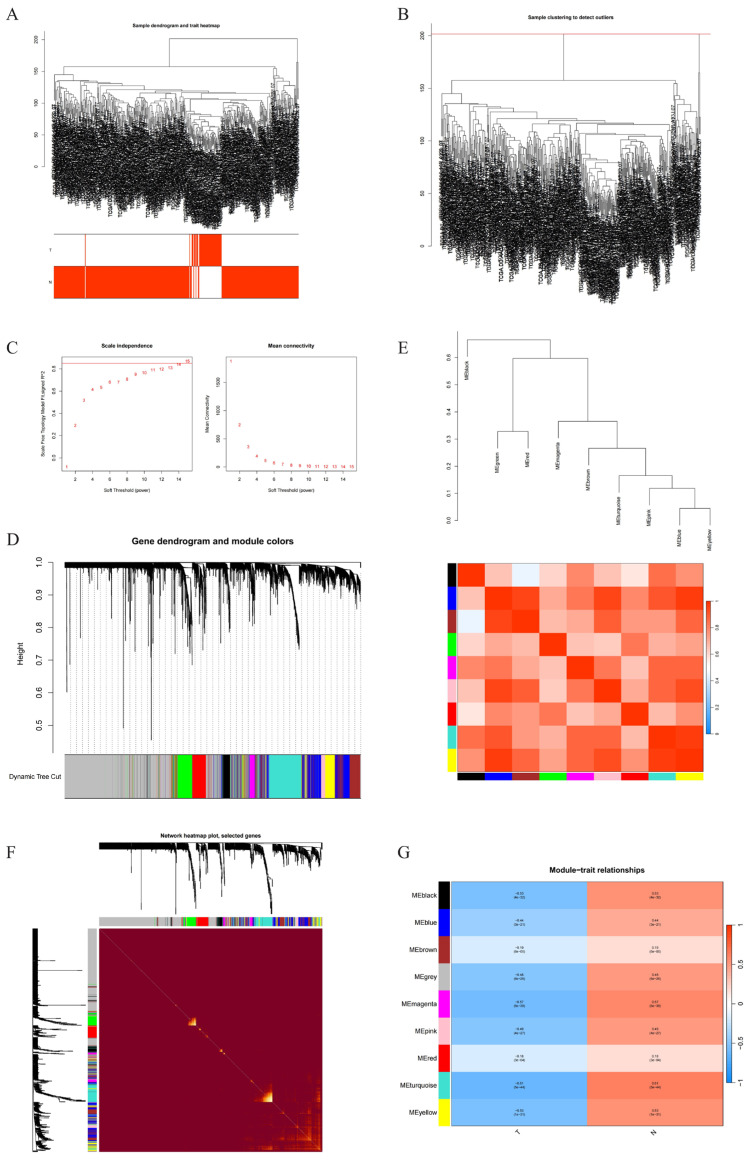
WGCNA network construction. (**A**) Sample clustering dendrogram. (**B**) Soft threshold selection (β = 10) and scale-free topology R^2^. (**C**) Gene clustering dendrogram. (**D**) Initial and merged module identification. (**E**) Module eigengene correlation heatmap showing positive (red) and negative (blue) associations. (**F**) Module eigengene clustering dendrogram. (**G**) Module-trait correlation heatmap displaying positive (red) and negative (blue) relationships.

Transcriptomic data for liver hepatocellular carcinoma (LIHC) were downloaded from the Cancer Genome Atlas (TCGA) website. A total of 50 normal samples and 374 hepatocellular carcinoma samples were selected for hierarchical clustering analysis, excluding obviously abnormal samples by setting thresholds, as shown in Figure 2A,B. When R^2^ > 0.85 and average connectivity was high, soft threshold was set at 15, as shown in Figure 2C. Through significance screening with *p*-value < 0.05, 9 modules were determined for further study. Initial and merged modules are finally displayed under clustering tree (Figure 2D). Next, correlations between modules were examined, showing no significant associations between them (Figure 2E). Intra-modular transcriptional correlation analysis demonstrated the reliability of module assignments, showing no substantial associations between modules (Figure 2F). Correlations between ME values and clinical features were applied to explore associations between modules and clinical symptoms. Among these, MEturquoise module showed positive correlation with normal samples (R = 0.61, *p* = 5 × 10^−44^) and negative correlation with hepatic cancer (LIHC) (R = −0.61, *p* = 5 × 10^−44^) (Figure 2G). Therefore, it was identified as a clinically significant module. Cytoscape (3.10.3) was used for gene network analysis, with cytoHubba plugin screening hub genes, defining top 30 ranked genes as hub genes.

### 2.4. Core Gene Screening and Clinical Survival Prognosis Analysis

**Figure 3:**
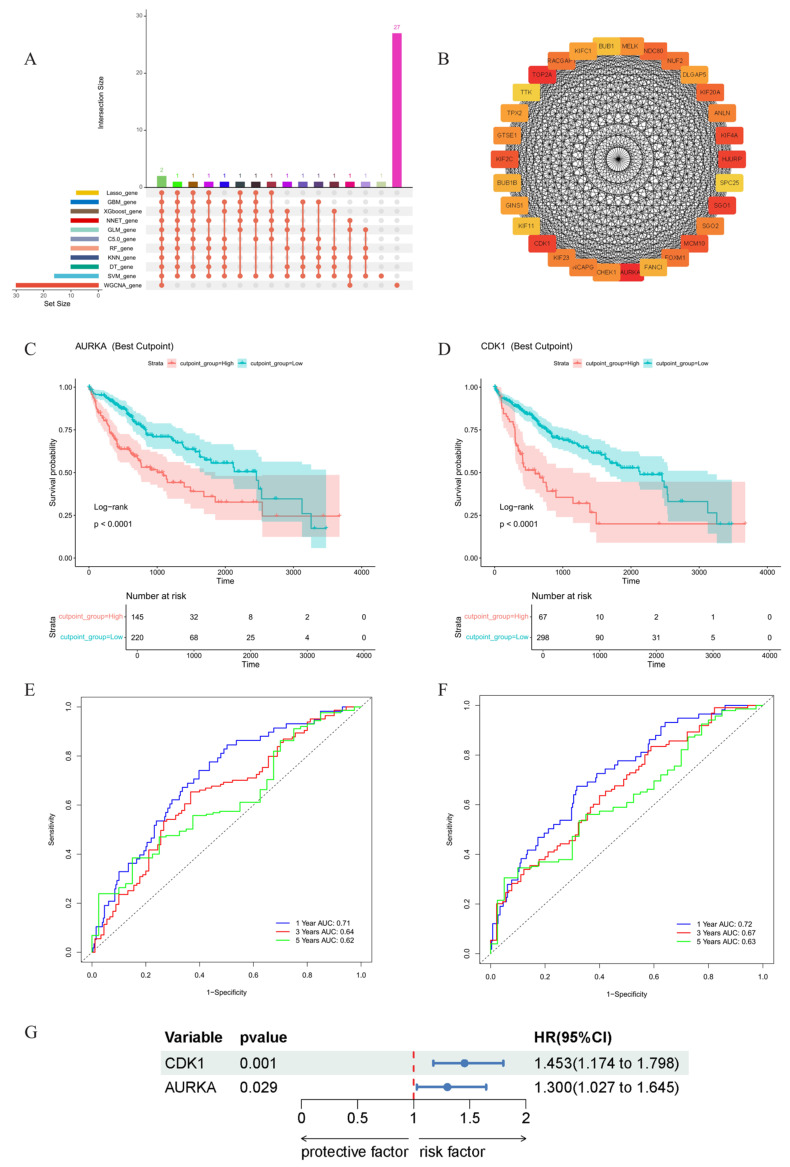
(**A**) Gene intersection between 10 machine learning algorithms and top 30 WGCNA hub genes with high phenotypic correlation. (**B**) PPI network of top 30 turquoise module genes showing highest WGCNA correlation. (**C**) AURKA Kaplan–Meier survival curves in hepatic cancer patients. (**D**) CDK1 Kaplan–Meier survival analysis in hepatic cancer patients. (**E**) AURKA gene ROC curve analysis in hepatic cancer patients. (**F**) CDK1 gene ROC curve analysis in hepatic cancer patients. (**G**) Univariate Cox regression results.

Core genes were determined by intersecting genes identified by 10 machine learning algorithms with 30 hub genes highly correlated with phenotypes from WGCNA, identifying AURKA and CDK1 as core genes (Figure 3A,B). Kaplan–Meier survival analysis was performed on identified core genes. As shown in Figure 3C,D, red lines represent survival of high gene expression groups, blue lines represent low expression groups, with *p* < 0.05 in lower left corner indicating gene expression correlation with survival. Lower graphs show risk tables with high/low expression groups based on gene expression levels, with numbers representing surviving patients over time. Both core genes showed *p* values < 0.05, with red lines representing high expression groups showing lower survival rates than low expression groups, indicating significant correlation between high expression of both core genes and shortened hepatic cancer survival time. ROC curve analysis was subsequently performed to evaluate gene performance in 1-year, 3-year, and 5-year survival prediction through time-series AUC. Model prediction values were all >0.7 within the first year, and >0.6 for three to five years thereafter, demonstrating model prediction reliability (Figure 3E,F). Univariate Cox regression analysis similarly indicated that AURKA and CDK1 gene expression are risk factors affecting patient survival (Figure 3G).

### 2.5. Molecular Docking Results

**Figure 4:**
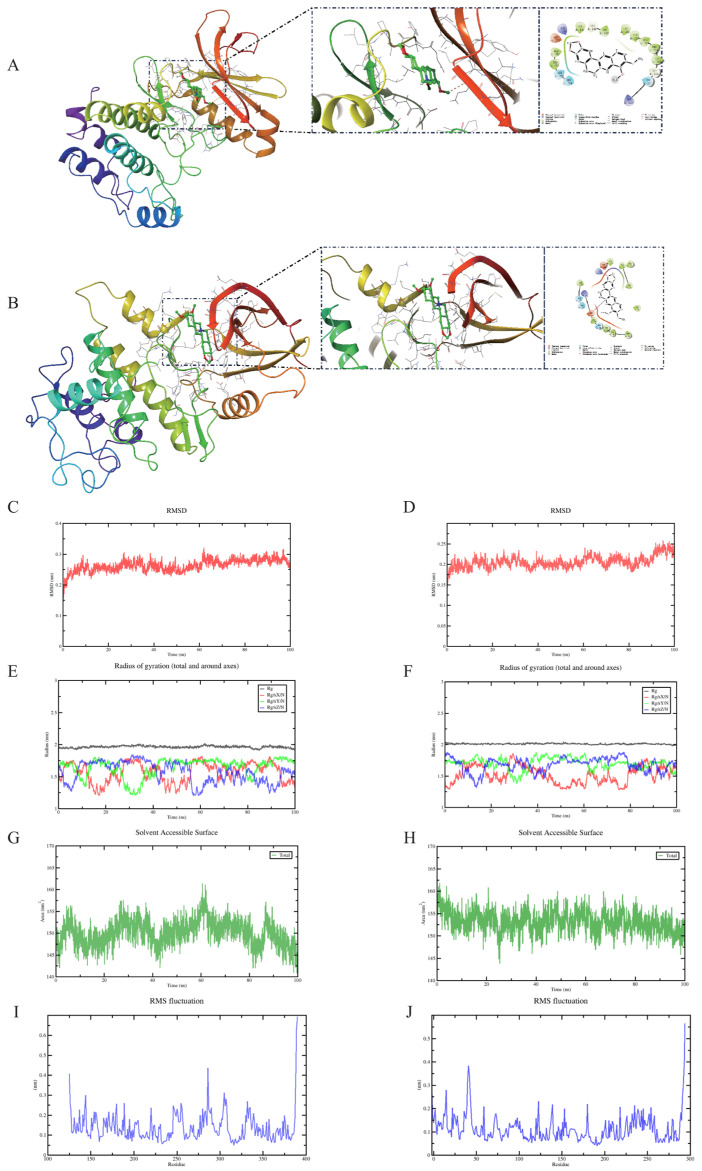
Berberine molecular docking and structural analysis with AURKA and CDK1. (**A**) Berberine-AURKA docking configuration. (**B**) Berberine-CDK1 docking configuration. (**C**) RMSD analysis for berberine-AURKA complex. (**D**) RMSD analysis for berberine-CDK1 complex. (**E**) Radius of gyration for berberine-AURKA complex. (**F**) Radius of gyration for berberine-CDK1 complex. (**G**) SASA measurements for berberine-AURKA complex. (**H**) SASA measurements for berberine-CDK1 complex. (**I**) Amino acid backbone RMSF in berberine-AURKA complex. (**J**) Amino acid backbone RMSF in berberine-CDK1 complex.

Binding energies lower than −5 kcal/mol indicate high receptor–ligand binding affinity [23]. Molecular docking results revealed that berberine exhibits high binding affinity for both AURKA and CDK1, with binding energies of −8.2 kcal/mol for AURKA and −8.4 kcal/mol for CDK1. The lowest energy binding conformations were visualized using PyMOL 2.3.0. The interaction between berberine and AURKA is depicted in Figure 4A. Berberine forms one hydrogen bond with the LYS residue of CDK1 (Figure 4B). These findings indicate that berberine may exert its anti-cancer effects through direct interactions with these targets, highlighting their significance in elucidating the mechanism of berberine in the treatment of hepatic cancer.

### 2.6. Molecular Dynamics Simulation of Protein-Ligand Complexes

Root mean square deviation (RMSD) trends indicate whether complexes achieve stability during MD simulation, with lower RMSD values meaning greater stability. RMSD fluctuation values for complexes formed by AURKA and CDK1 with berberine stabilized after 10 ns, indicating proteins reached stable states after ligand binding (Figure 4C,D). This indicates strong binding within complexes, emphasizing stability of binding interactions between berberine and AURKA and CDK1 [24]. Further analysis revealed that radius of gyration (Rg) values (Figure 4E,F) and solvent accessible surface area (SASA) (Figure 4G,H) of complex systems showed slight fluctuations during motion, indicating conformational changes in small molecule-target protein complexes during motion. Root mean square fluctuation (RMSF) can indicate flexibility of amino acid residues in proteins. As shown in Figure 4I,J, RMSF values for complex systems were generally low, with only C-terminal RMSF being higher, possibly representing flexible regions of proteins. Therefore, their flexibility was low with high stability. In conclusion, complex systems showed stable binding with good small molecule–target protein binding interactions.

### 2.7. GO and KEGG Analysis Results

**Figure 5:**
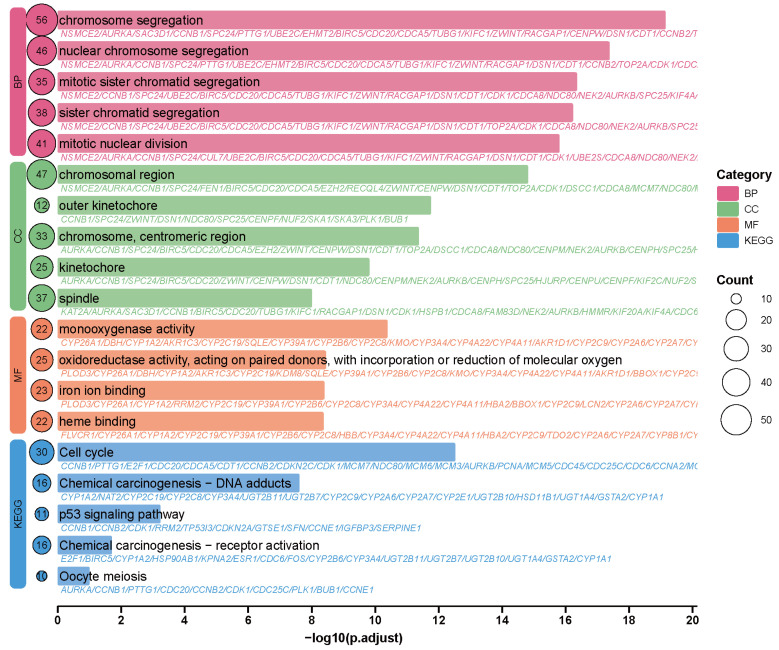
GO enrichment analysis (BP, CC, MF) and KEGG pathway significance.

Gene Ontology (GO) enrichment analysis indicated that berberine exerts anti-hepatocellular carcinoma effects primarily by influencing cell cycle progression, chromosome segregation, and mitosis (Figure 5). Key biological processes such as chromosome segregation and mitotic nuclear division were significantly enriched. These findings align with reports that AURKA and CDK1—critical regulators of mitosis—are overexpressed in hepatocellular carcinoma and linked to poor prognosis [25]. Berberine likely impedes mitotic progression by affecting AURKA, which phosphorylates CDK1 to activate the CDK1–Cyclin B complex and thus override the G2/M checkpoint [26], facilitating centrosome maturation and spindle assembly. Enrichment of cellular components including chromosomal regions, spindles, and kinetochores suggests that berberine induces chromosomal missegregation by disrupting mitotic apparatuses, leading to cell cycle arrest or apoptosis. Molecular function analysis highlighted oxidoreductase and monooxygenase activities, implying berberine may also alter oxidative metabolism, such as arachidonic acid pathways, to enhance oxidative stress in cancer cells [27]. Kyoto Encyclopedia of Genes and Genomes (KEGG) pathway analysis further confirmed the central role of the cell cycle pathway. CDK1 and AURKA serve as key nodes, regulating G2/M transition and centrosome separation, respectively. Inhibiting CDK1 induces G2/M arrest, while AURKA suppression disrupts spindle formation and chromosome segregation, reducing phosphorylation of PLK1 and CCNB1 [28]. Additionally, enrichment of the p53 pathway suggests another mechanism: berberine promotes AMPK-mediated mitochondrial/caspase-dependent apoptosis and may enhance p53-mediated death signaling in hepatocellular carcinoma [29].

## 3. Discussion

This study provides comprehensive computational evidence that berberine exerts anti-hepatocellular carcinoma effects through dual targeting of AURKA and CDK1, two critical cell cycle regulators frequently overexpressed in hepatocellular carcinoma. Our findings are consistent with and extend the growing body of literature on the multi-target anti-cancer properties of this natural compound. A recent comprehensive review systematically outlined berberine’s capacity to inhibit tumor growth and metastasis across various cancer types, underscoring its potential as a safe and useful adjuvant in oncology [30]. The consistent identification of AURKA and CDK1 across multiple machine learning algorithms—and their validation as hub genes by WGC-NA—strengthens the reliability of our findings, an approach increasingly recognized in computational drug discovery for natural products [31]. Molecular docking revealed high-affinity interactions between berberine and both targets, with favorable binding energies (−8.2 and −8.4 kcal/mol), while the 100 ns MD simulations confirmed stable binding modes and sustained affinity—a critical validation step in computational drug discovery [24]. Pathway enrichment analysis revealed that berberine’s effects extend beyond direct cell cycle inhibition to include p53 pathway activation and oxidative stress modulation. This multi-pathway engagement underscores berberine’s potential as a multi-target agent, capable of disrupting several oncogenic processes simultaneously. Notably, a very recent study demonstrated that berberine sensitizes liver cancer cells to sorafenib by inducing SETDB1/NQO1/p53-dependent ferroptosis and genomic instability [32], providing independent validation of berberine’s involvement in p53-related mechanisms. Furthermore, our results gain additional support from research showing berberine’s synergistic effects with other chemotherapeutic agents, such as its ability to increase the killing effect of pirarubicin on HCC cells by inhibiting the ATG4B-autophagy pathway [33]. This aligns with our network-based identification of cell cycle and apoptosis-related pathways, suggesting that berberine’s core function in HCC may involve sensitizing cancer cells to various forms of cellular stress and damage. Importantly, the central role of AURKA and CDK1 in hepatocellular carcinoma is corroborated by independent clinical bioinformatics analyses which also identified these kinases as prognostic biomarkers in HBV-related HCC [25].

Our integrated computational approach—combining machine learning, WGCNA, molecular docking, and MD simulations—represents a powerful strategy for elucidating the mechanisms of natural products, particularly valuable for compounds like berberine that exhibit polypharmacological effects [31]. The identification of AURKA and CDK1 as primary targets opens new avenues for berberine optimization and combination therapy development. For example, combining berberine with existing CDK1 or AURKA inhibitors may enhance therapeutic efficacy while minimizing adverse effects—a strategy worth exploring in preclinical models. Additionally, berberine’s documented ability to suppress metastasis and recurrence of hepatocellular carcinoma by targeting circulating tumor cells [34], further supports its potential clinical utility beyond primary tumor growth inhibition. Despite the computational rigor of our findings, limitations include reliance on public databases and the absence of experimental validation. Future studies should include in vitro kinase assays, cellular proliferation studies, and in vivo xenograft models to confirm berberine’s direct inhibition of AURKA and CDK1. In summary, this study not only elucidates a novel dual-target mechanism of berberine in HCC but also demonstrates the power of integrative computational biology in bridging the gap between traditional medicine and modern molecular oncology. The convergence of our computational predictions with recent experimental findings in the literature strengthens the case for continued investigation of berberine as a promising therapeutic agent in liver cancer.

## 4. Materials and Methods

### 4.1. Data Platforms and Software Tools

This study employed bioinformatics methods to analyze liver cancer differential genes and utilized databases to search traditional Chinese medicine names and active components. The data platforms and software tools used are listed in Table 1.

### 4.2. Data Sources

Transcriptomic datasets for liver hepatocellular carcinoma (LIHC) alongside corresponding patient clinical information were obtained from the Cancer Genome Atlas (TCGA) platform. UCSC Xena platform was utilized to acquire supplementary TCGA tumor transcriptomic data for gene expression analysis and survival analysis. SwissTargetPrediction and PharmMapper platforms were employed to predict berberine’s corresponding targets.

### 4.3. Analysis Methods and Workflow

#### 4.3.1. Differential Gene Analysis

The limma package in R language version 4.4.3 was utilized to examine hepatic cancer transcriptomic sequencing data for obtaining differentially expressed genes (DEGs) and generating volcano plots [35]. To ensure statistical significance and biological relevance of DEGs, thresholds were established as absolute log2 fold change (FC) > 1 and *p*-value < 0.05. Genes meeting these criteria were considered significant. Finally, volcano plots generated using the ggplot2 package were employed to visualize DEG distribution [36].

#### 4.3.2. Potential Targets of Berberine Against Liver Cancer

Through detailed retrieval of the PubChem database, berberine’s chemical information including SMILES notation was obtained. SMILES notation was subsequently uploaded to SwissTargetPrediction and PharmMapper platforms, which effectively identify potential drug targets by analyzing compound chemical structure combined with existing target information. Merging target data from both platforms ensured completeness of potential drug targets. To explore relationships between berberine’s predicted targets and hepatic cancer-related genes, Venn diagrams were constructed. These diagrams intuitively represented intersections and differences between datasets. By comparing DEGs in hepatic cancer with berberine’s predicted targets, overlapping targets were identified. These targets, involved in both hepatic cancer pathology and berberine’s pharmacological effects, were considered potential targets of berberine against hepatic cancer [37].

#### 4.3.3. Screening Key Targets of Berberine Against Liver Cancer Through Machine Learning

For identifying berberine’s critical targets in hepatocellular carcinoma, an ensemble of ten distinct machine learning methodologies was implemented: C5.0, Decision Tree, Gradient Boosting Machine (GBM), Generalized Linear Model (GLM), K-Nearest Neighbors (KNN), Least Absolute Shrinkage and Selection Operator (LASSO), Neural Network (NNET), Random Forest (RF), Support Vector Machine (SVM), and Extreme Gradient Boosting (XGBoost). These algorithms provided multi-angle screening and validation of targets, ensuring result robustness and reliability [38]. LASSO was constructed using the glmnet package, utilizing L1 regularization to compress unimportant feature coefficients to zero, simplifying the model and combining K-fold cross-validation to optimize regularization parameter λ to prevent overfitting. Decision Tree and C5.0 recursively split feature space based on information gain or gain ratio, screening high-contribution targets. GBM and XGBoost improved feature selection accuracy through iterative optimization of weak learners, combined with regularization and parallel computing. GLM fitted feature relationships through maximum likelihood estimation, combined with regularization for target screening. KNN evaluated target similarity based on distance metrics, identifying key features through nearest neighbor voting. NNET learned complex nonlinear relationships through multi-layer neural networks, combined with dropout to prevent overfitting. SVM utilized maximum margin hyperplanes and kernel functions for nonlinear classification, with SVM-RFE recursively eliminating low-weight features and cross-validation RMSE ensuring prediction accuracy. RF was optimized through the caret package, using incremental mean squared error to evaluate target importance, with higher values indicating greater contribution. Finally, by integrating cross-targets from ten algorithms, combined with feature importance assessment and cross-validation, we determined berberine’s core targets in liver cancer, providing a reliable foundation for subsequent research [39].

#### 4.3.4. Construction of Weighted Gene Co-Expression Network to Identify Key Genes

We performed weighted gene co-expression network analysis using WGCNA package (version 1.72) in R to investigate gene co-expression patterns and phenotype associations within the training dataset. The top 7000 genes ranked by median absolute deviation (MAD) were selected for network construction. Soft threshold determination utilized the pickSoftThreshold function with correlation coefficient R^2^ > 0.85 to establish scale-free topology. Module identification employed dynamic tree cutting with minimum module size of ninety genes. Module–phenotype correlations were calculated and visualized through heatmaps to assess association strength with disease characteristics. The module exhibiting strongest phenotypic correlation was designated as the key module. Network visualization and hub gene identification were conducted using Cytoscape (version 3.10.3) with cytoHubba plugin, ranking network nodes to identify the top 30 hub genes based on connectivity scores.

#### 4.3.5. Core Gene Screening and Clinical Survival Prognosis Analysis

Core genes were identified through intersection analysis of candidates from ten machine learning approaches and highly correlated hub genes derived from WGCNA. Survival analysis was conducted using Kaplan–Meier methodology via R packages survival and survminer, assessing relationships between gene expression (categorized by optimal cutoffs and median values) and patient outcomes. Performance evaluation employed ROC curve analysis through the timeROC package, calculating time-dependent AUC values for 1-, 3-, and 5-year survival predictions. Individual gene impact on patient prognosis was assessed via univariate Cox regression using the coxph function. Regression outputs were processed through summary (cox) to extract coefficients, from which hazard ratios (HR) and corresponding 95% confidence intervals were derived. Visual representation of univariate Cox regression results was achieved using forestploter package to generate forest plots.

#### 4.3.6. Molecular Docking

Target protein and berberine 3D structures were retrieved from Protein Data Bank (http://www.rcsb.org/, accessed on 8 July 2025) and PubChem databases. Molecular and protein structures underwent preprocessing via AutoDock Tools 1.5.6, including dehydration, hydrogenation, and charge computation. Molecular docking was performed using AutoDock to determine minimal binding affinity for drug–target interactions. Structural visualization was accomplished using PyMOL version 2.1.1 [40].

#### 4.3.7. Molecular Dynamics Simulation

Molecular dynamics simulations spanning 100 ns were conducted using GROMACS 2025 software on protein–ligand complexes. Force field parameterization employed CHARMM36 for protein structures and GAFF2 for ligand topology generation [41]. The complexes were positioned within cubic simulation boxes under periodic boundary conditions, with TIP3P water molecules filling the remaining space to create a 1.2 nm boundary buffer. Electrostatic calculations utilized Particle Mesh Ewald (PME) methodology combined with Verlet integration algorithms. System equilibration involved two sequential phases as follows: isothermal-isochoric (NVT) and isothermal-isobaric (NPT) ensembles, each comprising 100,000 steps with 0.1 ps coupling time constants over 100 ps intervals. Production simulations proceeded for 5,000,000 integration steps using 2 fs timesteps, accumulating 100 ns of trajectory data under controlled conditions (310 K temperature, 1 bar pressure) [42]. Trajectory analysis employed GROMACS utilities to compute root mean square deviation, root mean square fluctuation, radius of gyration, solvent-accessible surface area, principal component analysis, and free energy landscape mapping through covariance matrix evaluation.

#### 4.3.8. GO and KEGG Analysis

Functional enrichment analysis was conducted on differentially expressed genes from hepatic malignancies using clusterProfiler (v4.10.1) for Gene Ontology and KEGG pathway assessment. This R-based tool facilitates multi-species genomic analysis of protein-coding and non-coding transcripts. Annotations utilized GO and KEGG reference databases, with statistical evaluation via Benjamini–Hochberg-adjusted hypergeometric testing (corrected *p* < 0.05) to control false discovery rates. Hypergeometric distribution calculated gene set occurrence probabilities within functional categories. SRplot generated visualizations showcasing the ten most significantly enriched terms across cellular components, molecular functions, biological processes, and metabolic pathways.

## 5. Conclusions

This study employed an integrated approach combining transcriptomic analysis, multiple machine learning algorithms, WGCNA co-expression network analysis, and molecular simulation techniques. Additionally, this study systematically reveals the mechanism of berberine against hepatocellular carcinoma (HCC). Transcriptomic analysis identified 531 differentially expressed genes, and intersection with 173 potential targets of berberine yielded 17 common candidates. Ten machine learning methods consistently identified AURKA and CDK1 as key targets, which were further confirmed as hub genes by WGCNA. Survival analysis indicated that high expression of both genes was significantly associated with poor patient prognosis (*p* < 0.05). Molecular docking demonstrated strong binding affinity of berberine to AURKA (−8.2 kcal/mol) and CDK1 (−8.4 kcal/mol), and 100 ns molecular dynamics simulations verified the stability of the complexes. Functional enrichment analysis suggested that berberine exerts its anti-HCC effects mainly by interfering with cell cycle progression, chromosome segregation, and the p53 signaling pathway, indicating that its therapeutic effect is achieved through synergistic targeting of AURKA and CDK1. These findings provide molecular insights into the mechanism of berberine and lay a foundation for its subsequent experimental validation and clinical development as an adjunctive therapy for liver cancer.

## Figures and Tables

**Table 1:** Data platforms and software tools.

Platform/Software Name	URL	Date of Visit
The Cancer Genome Atlas (TCGA)	https://portal.gdc.cancer.gov/	Accessed 8 July 2025
UCSC Xena Data Platform	https://xena.ucsc.edu/	Accessed 8 July 2025
GeneCards Database	https://www.genecards.org/	Accessed 10 July 2025
PubChem Database	https://pubchem.ncbi.nlm.nih.gov/	Accessed 8 July 2025
Cytoscape 3.10.3	https://cytoscape.org/	Accessed 10 July 2025
PharmMapper Database	https://www.lilab-ecust.cn/pharmmapper/	Accessed 8 July 2025
SwissTargetPrediction Database	http://swisstargetprediction.ch/	Accessed 8 July 2025

## Data Availability

The original contributions presented in this study are included in the article. Further inquiries can be directed to the corresponding author.
